# Prevalence of asthma and allergies among children in the United Arab Emirates: A cross-sectional study

**DOI:** 10.1016/j.waojou.2021.100588

**Published:** 2021-10-06

**Authors:** Nassem Mohamed Ibrahim, Fatima Ibrahim Almarzouqi, Fatima Abdulla Al Melaih, Hisham Farouk, Mohamed Alsayed, Fatma Mohamed AlJassim

**Affiliations:** a Ministry of Health and Prevention / Primary Health Care Department, Dubai, United Arab Emirates; b AstraZeneca Gulf, United Arab Emirates; c Latifa Women & Children Hospital, Dubai, United Arab Emirates

**Keywords:** Asthma, Allergic rhinitis, Atopic dermatitis, ISAAC, UAE

## Abstract

**Background:**

Asthma, allergic rhinitis, and atopic dermatitis are the most common chronic inflammatory disorders in children worldwide. These conditions place a significant burden on the healthcare system due to their multiple related complications and the necessity of hospital visits. This study aimed to evaluate the prevalence and severity of asthma and allergic diseases among school children aged 6–7 years and 13–14 years in Dubai and the Northern Emirates, United Arab Emirates (UAE).

**Patients and methods:**

This was a multicenter, cross-sectional study that recruited children from March to June 2019 via school class registers in Dubai and the Northern Emirates, UAE. The Arabic and English versions of the International Study of Asthma and Allergies in Childhood (ISAAC) core questionnaires on asthma, allergic rhinitis, and atopic dermatitis were completed by parents or legal guardians of children aged 6–7 years, and by the children themselves in those aged 13–14 years.

**Results:**

In this study, we included 3436 children (1944 children aged 6–7 years and 1793 children aged 13–14 years). We estimated the prevalence of asthma (11.9%), wheezing (44.2%), allergic rhinitis (46.5%), hay fever (22.1%), and atopic dermatitis (12.9%) in the 6- to 7-year-old group. In the 13- to 14-year-old group, the prevalence was 9.8%, 33.1%, 51.3%, 19.9%, and 14.6%, respectively. The prevalence of any history of asthma was higher in boys than girls in the 6- to 7-year-old group (13.9% vs. 10%) and in the 13- to 14-year-old group (11.2% vs. 8.7%). In the 6- to 7-year-old group, the highest prevalence of asthma, sneezing, and atopic dermatitis was observed in Dubai, Ajman, and Umm Al Quwain, respectively. In the 13- to 14-year-old group, the highest prevalence of asthma was observed in Ras Al Khaimah, and the highest prevalence of sneezing and atopic dermatitis was observed in Sharjah.

**Conclusion:**

We found that the prevalence of asthma, allergic rhinitis, and atopic dermatitis in the UAE is comparable to that in neighboring countries; the prevalence of asthma, wheezing, and hay fever was higher in the 6- to 7-year-old group, while in the 13- to 14-year-old group, the prevalence of allergic rhinitis and atopic dermatitis was higher. Overall, the prevalence of any history of asthma was highest in Ras Al Khaimah, followed by Dubai, and lowest in Ajman. Our findings suggest that allergic disorders represent a healthcare burden in the UAE and more efforts are needed to organize nationwide campaigns to detect undiagnosed children to overcome the burden caused by these conditions.

## Introduction

Asthma, allergic rhinitis, and atopic dermatitis are the most common chronic inflammatory disorders in children worldwide.^1^ Asthma is characterized by various respiratory symptoms, including respiratory airflow limitations, dyspnea, wheeze, chest tightness, and cough that vary over time and intensity.^2^ Allergic rhinitis is considered an IgE-mediated disorder caused by exposure of the nasal mucosa to allergens.^3^ This leads to rhinorrhea, itching, airflow obstruction, sneezing, and sleep disturbance.^4^ Atopic dermatitis is closely associated with asthma and allergic rhinitis. Asthma, allergic rhinitis, and atopic dermatitis place a significant burden on the healthcare system due to their multiple related complications and the necessity of hospital visits.^5^^,^^6^

The etiology of asthma remains mostly unknown; nevertheless, asthma is known to be a heterogeneous and multifactorial disorder, which has a combination of non-modifiable (eg, heredity and sex) and modifiable (eg, environmental) risk factors.^7^ Lifestyle and genetic factors are also affecting the prevalence of allergic rhinitis and other allergic conditions.^8^^,^^9^ Perennial indoor allergens, seasonal pollens, and molds are the most common allergens that cause allergic rhinitis.^10^ Arab incense (bokhor) contributes to the prevalence and severity of asthma and allergic rhinitis.^11^ In the United Arab Emirates (UAE), a study showed that adolescents with tobacco exposure at home were more likely to report asthma, wheeze, and dry cough.^12^

It was thought that the prevalence of these diseases was higher in developed countries. However, over the past 2 decades, the global reports of the International Study of Asthma and Allergies in Childhood (ISAAC) demonstrated that the prevalence of these conditions was comparable or even higher in some developing countries than in developed ones.^13^, ^14^, ^15^ In the Gulf region, the prevalence of asthma in the UAE ranged between 6% and 13% in children aged 6–14 years. Moreover, 7.4% of the UAE general population have asthma compared with 6.4% in Kuwait, 4.8% in Oman, and 3.6% in Saudi Arabia.^16^^,^^17^ A recent systematic review conducted by Al-Herz et al demonstrated that the prevalence of allergic rhinitis in a sample of 397 children aged 6–9 years in the UAE was 8.1%.^18^ The prevalence of any history of wheeze, and wheeze during the last year, were reported to be influenced by sex, with a higher prevalence in male than female individuals in Kuwait.^19^ Interestingly, in the UAE, a large cross-sectional study demonstrated that the prevalence of asthma in patients with allergic rhinitis was 3 times higher than that in patients without allergic rhinitis (23.8% and 7.5%, respectively).^20^

Nevertheless, there is still a scarcity of data on the prevalence and/or distribution of asthma and allergies in UAE. Although some data have been published from Middle Eastern countries that participated in ISAAC, the sample size was small and only involved children aged 13–14 years. Therefore, our study aimed to describe the prevalence and severity of asthma and allergic diseases among children aged 6–7 years and 13–14 years in Dubai and the Northern Emirates.

## Subjects and methods

This study was reported in accordance with the guidelines of Strengthening the Reporting of Observational Studies in Epidemiology (STROBE) for cross-sectional design.^21^ The study was approved by the Ministry of Health and Prevention (MOHP), UAE (Ref No. MOHAP/DXB-REC-6/FM/2019), and the parents/legal guardians of the participants provided signed written informed consent.

### Study design and setting

A multicenter, cross-sectional study (NCT03878680) that recruited children from March to June 2019 via school class registers in Dubai and the Northern Emirates, UAE. School children from Government and private schools in the targeted age groups were included. The ISAAC core questionnaires on asthma, allergic rhinitis, and atopic dermatitis were completed by parents or legal guardians of children in the 6- to 7-year-old group and by the children themselves in the 13- to 14-year-old group.^22^ To overcome translation problems and the need to describe symptoms verbally, children aged 13- to 14 years completed a video questionnaire. We validated the research instruments (video and written questionnaires) with the bronchial hyperreactivity challenge that was used in the study of Hong et al.^23^

### Inclusion and exclusion criteria

We included all children who met the following criteria: 1) children aged 6–7 years or 13–14 years residing in Dubai and the Northern Emirates upon completion of the questionnaire and 2) children who can understand the questions of the questionnaire themselves in the 13- to 14-year-old group. Written informed consent was obtained from the legal guardians of enrolled children. Children aged 13–14 years and unable to answer the questionnaire due to physical or mental disability or those whose parent/legal guardian refused to sign/return consent for both groups were excluded.

### Study questionnaire and data collection

The ISAAC questionnaire, validated by the World Health Organization (WHO), was used in Arabic and English versions.^22^ The Arabic version of the ISAAC questionnaire was adopted from Behbehan et al.^19^ The standard core questionnaire consists of wheezing, allergic rhinitis, atopic dermatitis, environmental questionnaire, and video questionnaire (only for the 13- to 14-year-old group). Questions regarding exposure to Arab “bokhor” and smoking were also included in the environmental questionnaire. By asking about cardinal symptoms, case definitions and severity were identified. The data collection date was recorded, and the study population was investigated from March to June before the summer months, as the summer months represent the main pollen period in the UAE. The demographic data were collected from the school registry (name of the school, date of birth, residence, ethnicity, and sex).

### Study variables

The primary outcomes of the present study were the prevalence of asthma, wheezing or whistling, and allergies (rhinitis and atopic dermatitis) in children aged 6–7 years and 13–14 years. The secondary outcomes of the present study included the severity of asthma, wheezing or whistling, and allergies (rhinitis and atopic dermatitis) during the 12 months before data collection.

For the assessment of wheezing or whistling, participants were asked to state if they ever had an episode of wheezing or whistling, as well as the frequency of wheezing or whistling episodes during the 12 months before data collection. The assessment of the severity of wheezing or whistling episodes was based on patient-reported limitation of exercise, speech limitation, and sleep disturbance due to wheezing or whistling episodes during the 12 months before data collection. The diagnosis of asthma was based on a self-reported lifetime occurrence of an asthma episode.

For identifying allergic rhinitis, participants were asked to state if they ever had an episode of 1 of the following symptoms: sneezing or runny/blocked nose not related to cold or flu; nose problem accompanied by itchy watery eyes; or hay fever. The assessment of the severity of allergic rhinitis was based on the self-reported limitation of daily activities due to nose problems during the 12 months prior to data collection.

Participants were also asked to report a lifetime occurrence of itchy rash that lasted for at least 6 months and atopic dermatitis. The severity of atopic dermatitis was based on the self-reported sleep disturbance due to itchy rash during the past 12 months before data collection.

### Sample size

With a sample size of at least 1500 school children in each of the 2 age categories, the study was able to estimate the prevalence of asthma in each age group within a margin of error of no more than 2.5% using 95% confidence intervals.

### Selection of study subjects

To overcome potential bias, a two-step stratified cluster random sampling was used separately for each age group. For each age group, the Ministry of Education (MOE) and Knowledge & Human Development Authority (KDHA) provided the total number of school children stratified by sex, district, and type of school (public vs private). The first step was to randomly select several schools in each district/sex/type of school strata for each age group. The second step was to select several school children from the schools chosen randomly. The number of schools in each stratum and the number of school children randomly selected from each school was based on the strata size and school size, respectively. Once the number was chosen, then school selection was performed within each stratum using complete randomization.

### Statistical analysis

Descriptive statistics in the form of mean and standard deviation were used for continuous variables (eg, age). Frequency distribution was used for categorical variables (eg, sex and nationality). Primary and secondary outcomes (such as asthma/wheezing, allergies, and the severity of symptoms) were summarized using frequency distributions and 95% confidence intervals (CIs). The Wald confidence interval was used unless cell counts fell below 5, in which case the Clopper–Pearson confidence interval was used. The overall prevalence was calculated for the 6- to 7- and 13- to 14-year-old groups, and then stratified by sex. Difference in the main outcomes between the 2 age groups and gender differences within each age group were assessed using the Chi-squared test (or Fisher's exact test when expected cell counts fell below 5). The association between variables and the primary outcome of any history of asthma was performed at the bivariate level using the chi-square tests for categorical variables and the independent *t*-test or Wilcoxon Rank Sum test (as a sensitivity analysis). The same associations were also assessed using bivariate logistic regression models to be able to build a multivariable model. The multivariate logistic regression model included all the variables with a p-value of 0.20 or less at the bivariate level. Potential collinearity between the independent variables were assessed using variance inflation factors (VIFs) and no collinearity was found (all VIFs were below 2). Unadjusted and adjusted odds ratios along with their 95% CIs were presented. Adjusted prevalence ratios were computed using the methods by Zhang and Yu^24^ and presented along with their 95% confidence intervals and p-values. Agreement between answers to the written and video questionnaires were summarized using percentage of concordant answers, percentage of agreement on positive and negative responses and the Kappa statistics. All analyses were performed using IBM-SPSS (version 25, USA). A p-value of 0.05 or less was considered statistically significant.

## Results

### Study participation

**Fig. 1:**
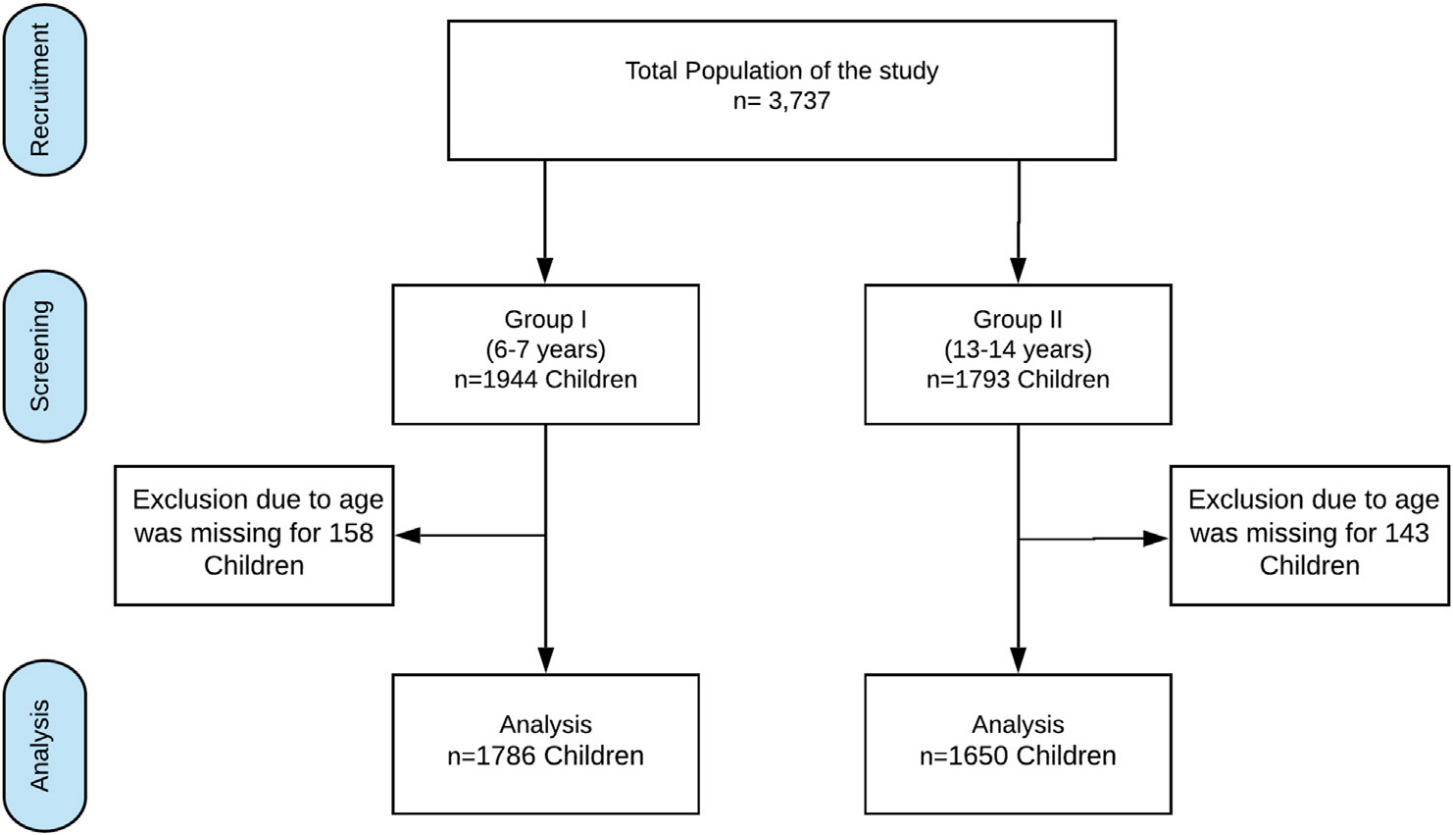
Flowchart of selection, screening, and analysis process

Data were collected from a total of 1944 children in the 6- to 7-year-old group and 1793 children in the 13- to 14-year-old group. Age data were missing for 158 children in the 6- to 7-year-old group and 143 in the 13- to 14-year-old group; thus, the number of children eligible for the analysis was 1786 children in the 6- to 7-year-old group and 1650 children in the 13- to 14-year-old group (Fig. 1).

### Characteristics of participants

The mean age of the 6- to 7- and 13- to 14-year-old groups was 6.8 ± 0.5 and 13.6 ± 0.5 years, respectively. More than half of the children in the 6- to 7- and 13- to 14-year-old groups were female (919 [51.7%] and 1000 [60.9%], respectively). The most frequently reported ethnicities in both groups were Arab and South Asian. The children's demographics are summarized in Table 1.

**Table 1:** The demographic characteristics, history of tobacco use, and history of exposure to incense of the study participants (n = 3436).

Variable	6–7 years		13–14 years	
	N	%	N	%
**Age (in years)**				
mean/SD	6.8	0.5	13.6	0.5
Median/(IQR)	6.7	(6.4, 7.1)	13.6	(13.2, 14.0)
**Gender**				
Female	919	51.7%	1000	60.9%
Male	859	48.3%	641	39.1%
Missing	8		9	
**Ethnicity**				
Arab	1044	60.6%	1014	62.0%
South Asian	526	30.5%	547	33.4%
Others	152	8.8%	75	4.6%
Missing	64		14	
**Type of School**				
Private	1369	76.7%	1049	63.6%
Public	417	23.3%	601	36.4%
**City of School**				
Ajman	176	9.9%	161	9.8%
Dubai	588	32.9%	571	34.6%
Fujairah	187	10.5%	241	14.6%
Ras Al Khaimah	176	9.9%	176	10.7%
Sharjah	615	34.4%	463	28.1%
Umm Al Quawain	44	2.5%	38	2.3%
**City of Residence**				
Abu Dhabi	1	0.1%	1	0.1%
Ajman	211	12.6%	169	10.5%
Dubai	538	32.0%	516	32.0%
Fujairah	136	8.1%	228	14.2%
Ras Al Khaimah	170	10.1%	147	9.1%
Sharjah	585	34.8%	509	31.6%
Umm Al Quawain	40	2.4%	41	2.5%
Missing	105		39	
**Father smoking (Yes)**	392	22.0%	354	21.5%
**Mother Smoking (No)**	28	1.6%	54	3.3%
**Number of people smoking in the house**				
None	607	66.4%	816	71.6%
1	247	27.0%	223	19.6%
2	35	3.8%	60	5.3%
More than 2	25	2.7%	40	3.5%
**Exposure to Incense**	738	42.5%	1168	71.2%
**Frequency of Incense exposure**				
No exposure	997	57.5%	473	28.8%
At least once a week	557	32.1%	877	53.4%
At least once a month	122	7.0%	206	12.6%
At least once a year	56	3.2%	82	5.0%
Missing-frequency of exposure	3	0.2%	3	0.2%
Missing exposure	51		9	

SD: Standard deviation

### History of tobacco and incense exposure

The percentage of fathers and mothers of children in the 6- to 7- and the 13- to 14-year-old groups who smoked were comparable. Approximately 30.8% of participants in the 6- to 7-year-old group and 24.9% in the 13- to 14-year-old group reported that 1 or 2 persons in the household smoked. Regarding exposure incense, in the 6- to 7-year-old group, 738 (42.5%) of the participants reported exposure. The percentage of participants reporting incense exposure was higher in the 13- to 14-year-old group (1168, 71.2%). Parents’ smoking status and exposure to incense are summarized in Table 1.

## Primary and secondary outcomes

### Asthma and wheezing

The prevalence of any history of wheezing or whistling was significantly higher in the 6- to 7-year-old group than in the 13- to 14-year-old group (44.2% vs. 33.1%, p < 0.001). There was no significant difference between boys and girls (44.4% vs. 44.0%, respectively, p = 0.861) in the 6- to 7-year-old group. In contrast, in the 13- to 14-year-old group, there was a higher percentage of boys than girls (38.2% vs. 29.9%, respectively, <0.001). Similarly, the prevalence of any history of wheezing or whistling in the past year was higher in the 6- to 7-year-old group than in the 13- to 14-year-old group (37.3% vs. 28.5%, p < 0.001), without any significant difference between boys and girls (36.9% vs. 37.5%, respectively, p = 0.107) in the 6- to 7-year-old group. However, the prevalence of any history of wheezing or whistling in the past year was higher in boys than in girls (32.4% vs. 25.9%, respectively, p = 0.005) in the 13- to 14-year-old group. The prevalence of ever asthma was 11.9% and 9.8% among the children aged 6–7 and 13–14 years, respectively. Male patients had significantly higher prevalence of ever asthma than female in the 6- to 7-year-old group (13.9% vs. 10%, p = 0.021). Concerning symptom severity, 8.2% and 7.8% of children aged 6–7 and 13–14 years experienced disturbed sleep due to wheezing more than once per week, respectively. Likewise, 5.1% and 15.5% of children aged 6–7 and 13–14 years experienced limited speech between breaths due to wheezing more than once per week, respectively. There were no significant differences between males and females concerning symptoms severity in the children aged 6–7 and 13–14 years (p > 0.05). Data regarding wheezing and asthma are summarized in Table 2, Table 3.

**Table 2:** The overall and sex-stratified prevalence of asthma and wheezing symptoms among children aged 6–7years old (n = 1778).

Variables		6–7 years (n = 1778)						p-value
		Overall		Female (919)		Male (= 859)		
		n (%)	95% CI	n (%)	95% CI	n (%)	95% CI	
Ever Wheezing or Whistling^a^		784 (44.2%)	(41.9%, 46.6%)	401 (44%)	(40.8%, 47.2%)	379 (44.4%)	(41.1%, 47.8%)	0.861
Past 12 months Wheezing or Whistling		661 (37.3%)	(35.1%, 40.0%)	342 (37.5%)	(34.4%, 40.7%)	315 (36.9%)	(33.7%, 40.2%)	0.790
Ever Asthma^a^		178 (11.9%)	(10.2%, 13.5%)	77 (10%)	(7.9%, 12.2%)	101 (13.9%)	(11.4%, 16.4%)	0.021^b^
Past 12 months chest wheezy during exercise		75 (4.4%)	NA	32 (3.6%)	NA	43 (5.2%)	NA	0.107
Past 12 months dry cough at night not related to cold or chest infection		473 (27.4%)	NA	242 (27.3%)	NA	230 (27.6%)	NA	0.856
		**n (%)**	**% among all those who answered the wheezing first question**	**n (%)**	**% among all those who answered the wheezing first question**	**n (%)**	**% among all those who answered the wheezing first question**	
**Past 12 months number of wheezing episodes**	none	380 (64.4%)	21.4%	207 (68.5%)	22.7%	170 (59.6%)	19.9%	0.025^b^
	1 to 3	178 (30.2%)	10.0%	83 (27.5%)	9.1%	95 (33.3%)	11.1%	0.123
	4 to 12	27 (4.6%)	1.5%	10 (3.3%)	1.1%	17 (6%)	2.0%	0.167
	more than 12	5 (0.8%)	0.3%	2 (0.7%)	0.2%	3 (1.1%)	0.4%	0.687
**Past 12 months, disturbed sleep due to wheezing**	never	446 (78%)	25.2%	234 (80.1%)	25.7%	209 (75.5%)	24.5%	0.178
	less than once/week	79 (13.8%)	4.5%	38 (13%)	4.2%	41 (14.8%)	4.8%	0.538
	one or more/week	47 (8.2%)	2.7%	20 (6.8%)	2.2%	27 (9.7%)	3.2%	0.209
Past 12-month, limit speech between breaths due to wheezing		32 (5.1%)	1.8%	13 (4%)	1.4%	19 (6.4%)	2.2%	0.177

NA: Not available, CI: Confidence Interval.

a Ever means lifetime occurrence.

b Significant difference at the 5% level

**Table 3:** The overall and sex-stratified prevalence of asthma and wheezing symptoms among children aged 13–14 years old (n = 1641).

Variables		13–14 years (n = 1641)						p-value
		Overall		Female (n = 1000)		Male (= 641)		
		n (%)	95% CI	n (%)	95% CI	n (%)	95% CI	
Ever Wheezing or Whistling^a^		545 (33.1%)	(30.9%, 35.4%)	298 (29.9%)	(27.0%, 32.7%)	244 (38.2%)	(34.4%, 42.0%)	<0.001^b^
Past 12 months Wheezing or Whistling		466 (28.5%)	(26.2%, 30.7%)	257 (25.9%)	(23.2%, 28.6%)	206 (32.4%)	(28.8%, 36.0%)	0.005^b^
Ever Asthma^a^		148 (9.8%)	(8.3%, 11.2%)	79 (8.7%)	(6.8%, 10.5%)	67 (11.2%)	(8.7%, 13.7%)	0.104
Past 12 months chest wheezy during exercise		307 (20.2%)	(18.2%, 22.2%)	185 (20.4%)	(17.8%, 23.0%)	119 (19.7%)	(16.5%, 22.8%)	0.729
Past 12 months dry cough at night not related to cold or chest infection		499 (32.9%)	(30.5%, 35.2%)	294 (32.6%)	(29.5%, 35.7%)	202 (33.1%)	(29.4%, 36.8%)	0.833
		**n (%)**	**% among all those who answered the wheezing first question**	**n (%)**	**% among all those who answered the wheezing first question**	**n (%)**	**% among all those who answered the wheezing first question**	
**Past 12 months number of wheezing episodes**	none	220 (50.6%)	13.4%	106 (44.2%)	10.6%	112 (58.3%)	17.5%	0.003^b^
	1 to 3	164 (37.7%)	10.0%	95 (39.6%)	9.5%	69 (35.9%)	10.8%	0.438
	4 to 12	37 (8.5%)	2.3%	31 (12.9%)	3.1%	5 (2.6%)	0.8%	<0.001^b^
	more than 12	14 (3.2%)	0.9%	8 (3.3%)	0.8%	6 (3.1%)	0.9%	0.903
**Past 12 months, disturbed sleep due to wheezing**	never	314 (76.4%)	19.2%	168 (73.4%)	16.9%	143 (79.9%)	22.4%	0.124
	less than once/week	65 (15.8%)	4.0%	41 (17.9%)	4.1%	24 (13.4%)	3.8%	0.218
	one or more/week	32 (7.8%)	2.0%	20 (8.7%)	2.0%	12 (6.7%)	1.9%	0.449
Past 12-month, limit speech between breaths due to wheezing		67 (15.5%)	4.1%	38 (15.5%)	3.8%	27 (14.5%)	4.2%	0.775

NA: Not available, CI: Confidence Interval.

a Ever means lifetime occurrence.

b Significant difference at the 5% level

### Allergic rhinitis

The prevalence of any history of sneezing or runny or blocked nose not related to a cold or flu was 46.5% in the 6- to 7-year-old group and 51.3% in the 13- to 14-year-old group. Regarding sneezing or runny or blocked nose not related to a cold or flu in the past 12 months, the prevalence was 42.6% in the 6- to 7-year-old group and 45.9% in the 13- to 14-year-old group, without any significant sex difference. In the 13- to 14–year-old group, the prevalence of nose problem accompanied by itchy watery eyes in the past 12 months was higher among females than males (15.1% vs. 11.5%, respectively, p = 0.042). The prevalence of any history of hay fever was 22.1% in the 6- to 7-year-old group and 19.9% in the 13- to 14-year-old group. Concerning symptoms severity, 36.8% and 20.4% of the children aged 6–7 and 13–14 years experienced moderate-to-severe nose problems interfering with daily activity, respectively. Data regarding reported allergic rhinitis are summarized in Table 4.

**Table 4:** The overall and sex-stratified prevalence of allergic rhinitis variables among the study participants (n = 3436).

Variables		6–7 years (n = 1778)						p-value	13–14 years (n = 1641)						*P*-value
		Overall		Female (919)		Male (= 859)			Overall		Female (n = 1000)		Male (n = 641)		
		n (%)	95% CI	n (%)	95% CI	n (%)	95% CI		n (%)	95% CI	n (%)	95% CI	n (%)	95% CI	
Ever problem with sneezing or runny/blocked nose not related to cold or flu^b^		815 (46.5%)	(44.2%, 48.9%)	409 (45.4%)	(42.2%, 48.7%)	402 (47.6%)	(44.3%, 51.0%)	0.360	841 (51.3%)	(48.9%, 53.7%)	496 (49.9%)	(46.8%, 53.1%)	340 (53.3%)	(49.4%, 57.2%)	0.188
Past 12 months problem with sneezing or runny/blocked nose not related to cold or flu		747 (42.6%)	(40.3%, 45.0%)	377 (41.9%)	(38.7%, 45.1%)	366 (43.4%)	(40.0%, 46.7%)	0.533	746 (45.9%)	(43.5%, 48.4%)	441 (44.9%)	(41.8%, 48.0%)	300 (47.5%)	(43.6%, 51.4%)	0.283
Past 12 months, nose problem accompanied by itchy watery eyes		139 (8%)	(6.7%,9.3%)	73 (8.2%)	(6.4%, 9.9%)	66 (7.9%)	(6.1%, 9.7%)	0.822	224 (13.8%)	(12.1%, 15.4%)	149 (15.1%)	(12.9%, 17.3%)	73 (11.5%)	(9.0%, 14.0%)	0.042^c^
Past 12 months, nose problem interfere with child daily activity	not at all	20 (14.7%)	NA	11 (15.3%)	NA	9 (14.1%)	NA	0.842	194 (26.0%)	11.9%^a^	117 (29.7%)	11.9%^a^	77 (29.1%)	12.2%^a^	0.860
	Little	66 (48.5%)		35 (48.6%)		31 (48.4%)		0.984	315 (42.2%)	19.4%^a^	185 (47.0%)	18.8%^a^	129 (48.7%)	20.4%^a^	0.664
	moderate	39 (28.7%)		21 (29.2%)		18 (28.1%)		0.893	125 (16.8%)	7.7%^a^	77 (19.5%)	7.8%^a^	47 (17.7%)	7.4%^a^	0.561
	a lot	11 (8.1%)		5 (6.9%)		6 (9.4%)		0.604	27 (3.6%)	1.7%^a^	15 (3.8%)	1.5%^a^	12 (4.5%)	1.9%^a^	0.647
Ever had hay fever		332 (22.1%)	(20.0, 24.2%)	161 (20.9%)	(18.0%, 23.8%)	170 (23.4%)	(20.3%, 26.5%)	0.249	303 (19.9%)	(17.9%, 22.0%)	180 (20.0%)	(17.4%, 22.6%)	122 (19.9%)	(16.7%, 23.1%)	0.963

a Percentage from those reporting at 12 months, NA: Not available, CI: Confidence Interval.

b Ever means lifetime occurrence.

c Significant difference at the 5% level

### Atopic dermatitis

The prevalence of any history of itchy rash that was present intermittently for at least 6 months was almost one-third in the 6- to 7-year-old group and one-quarter in the 13- to 14-year-old group. In terms of the past 12 months, the prevalence was 33.7% in the 6- to 7-year-old group and 23.4% in the 13- to 14-year-old group. This rash completely cleared during the past 12 months in 10.7% of affected children in the 6- to 7-year-old group and 58.3% in the 13- to 14-year-old group. The prevalence of any history of atopic dermatitis was comparable between both groups at 12.9% in the 6- to 7-year-old group and 14.6% in the 13- to 14-year-old group; however, in the 13- to 14-year-old group, the prevalence of any history of atopic dermatitis was higher in females than males (17% vs. 11.3%, respectively, p = 0.021). Concerning symptoms severity, 6.7% and 12.4% of children aged 6–7 and 13–14 years experienced an itchy rash that awoke them more than 1 per night, respectively. Reported results on possible atopic dermatitis allergies are summarized in Table 5.

**Table 5:** The overall and sex-stratified prevalence of atopic dermatitis allergies variables among the study participants (n = 3436).

Variables		6–7 years (n = 1778)						p-value	13–14 years (n = 1641)								p-value
		Overall		Female (919)		Male (= 859)			Overall		Female (n = 1000)			Male (n = 641)			
		n (%)	95% CI	n (%)	95% CI	n (%)	95% CI		n (%)	95% CI	n (%)	95% CI		n (%)	95% CI		
Ever had itchy rash which was coming and going for 6 months at least^a^		613 (35.1%)	(32.9%, 37.3%)	326 (36.0%)	(32.9%, 39.1%)	284 (34.1%)	(30.8%, 37.3%)	0.390	413 (25.2%)	(23.1%, 27.3%)	242 (24.3%)	(21.6%, 27.1%)		168 (26.5%)	(23.1%, 30.0%)		0.304
Past 12 months itchy rash which was coming and going for 6 months at least		589 (33.7%)	(31.5%, 35.9%)	311 (34.4%)	(31.3%, 37.5%)	275 (33.0%)	(29.8%, 36.2%)	0.540	383 (23.4%)	(21.3%, 25.4%)	221 (22.2%)	(19.6%, 24.8%)		159 (25.2%)	(21.8%, 28.5%)		0.121
Has the itchy rash affected fold of elbows, behind the knees, in front of the ankles, under the buttocks or around the neck, ears, or eyes?		158 (9.1%)	(7.8%, 10.6%)	82 (9.1%)	(7.2%, 11.0%)	76 (9.2%)	(7.2%, 11.1%)	0.970	186 (11.7%)	(10.1%, 13.2%)	125 (12.9%)	(10.8%, 15.0%)		59 (9.6%)	(7.3%, 11.9%)		0.047^b^
At what age did this itchy rash first occur	under 2	80 (35.2%)	NA	38 (33.0%)	NA	42 (37.5%)	NA	0.482									
	from 2 to 4	69 (30.4%)		42 (36.5%)		27 (24.1%)		0.042^b^									
	5 or more	78 (34.4%)		35 (30.4%)		43 (38.4%)		0.207									
	No occurrence/missing	1559		804		747											
Past 12 months child kept awake by itchy rash	Never	319 (82.0%)	NA	174 (82.5%)	Na	145 (81.5%)	NA	0.797	179 (58.3%)	NA	107 (56.9%)	NA		71 (61.2%)	NA		0.461
	<1 per night	44 (11.3%)		22 (10.4%)		22 (12.4%)		0.549	90 (29.3%)		54 (28.7%)			35 (30.2%)			0.787
	one or more per night	26 (6.7%)		15 (7.15%)		11 (6.2%)		0.715	38 (12.4%)		27 (14.4%)			10 (8.6%)			0.137
Ever had Eczema^a^		186 (12.9%)	(11.1%, 14.6%)	90 (12.1%)	(9.8%, 14.5%)	96 (13.8%)	(11.2%, 16.3%)	0.364	219 (14.6%)	(12.8%, 16.4%)	150 (17.0%)	(14.6%, 19.5%)		69 (11.3%)	(8.8%, 13.9%)		0.002^b^

NA: Not available, CI: Confidence Interval.

a Ever means lifetime occurrence.

b Significant difference at the 5% level

### Results of the video questionnaire (13- to 14-year-old group only)

The prevalence of any history of wheezing and wheezing in the last year was 17.1% and 12.6%, respectively. Approximately 19% and 15% reported any history of wheezing and wheezing in the last year during exercise, respectively. Any history of severe asthma and severe asthma in the last year were reported by 11.2% and 8.9%, respectively (eTable 1). Boys reported a higher prevalence of any history of wheezing during exercise (p = 0.001; eTable 2).

### Bivariate analysis

Several variables were significantly associated with any history of asthma. In terms of the 6- to 7-year-old group, any history of asthma was significantly higher among boys than among girls (13.9% vs 10.0%, p = 0.021), among those exposed to incense (16.7% vs. 8.7%, p < 0.001), and among those whose fathers were smokers (17.5% vs. 10.3%). Moreover, a significant association (p < 0.001) was observed between any history of asthma and any history of sneezing, any history of wheezing, any history of hay fever, and any history of atopic dermatitis. In terms of the 13- to 14-year-old group, any history of asthma was significantly higher among those of Arab ethnicity than among those of South Asian ethnicity (12.0% vs. 4.5%, p < 0.001), among those exposed to incense (10.9% vs. 7.0%, p = 0.018), among those whose fathers were smokers (13.1% vs. 8.9%, p = 0.023; eTable 3).

### Multivariate analysis

In both groups, most symptoms except for sneezing were still significantly associated with asthma. The results of the multivariate logistic regressions revealed that relative to the study's Asian population, Arab ethnicity was an independent predictor of asthma occurrence in the 6- to 7-year-old group (AROR = 2.167, 95% CI: 1.182–3.84.21, p = 0.0122) and in 13- to 14-year-old group (AROR = 2.7984, 95% CI: 1.6456–4.585.17, p ≤ 0.001). Moreover, in both groups, multivariate logistic regression analysis showed that tobacco and incense exposure were not associated with asthma (eTable 4).

### Concordance between written and video questionnaire responses

In terms of wheezing at rest, the concordance was reasonable for any history of wheezing and wheezing in the last year (72% and 74%, respectively) and high for nocturnal wheezing in the past year (93%). Such concordances did not change much and thus remained reasonable for any history of wheezing and wheezing in the last year at (70% and 71%, respectively) when the video outcome was considered positive for any wheeze in the 3 videos: at rest, during exercise, or at night. Moreover, the proportion of agreements were reasonable/high for negative responses but low for positive ones (eTable 5).

### Prevalence according to residency

In the overall population, the highest asthma prevalence was observed in Ras Al Khaimah (12.3%). Moreover, the highest prevalence of sneezing was observed in Sharjah (51.1%). While in Ajman, we found the highest prevalence of itchy rash (35.8%). In terms of atopic dermatitis, the same prevalence was observed in Dubai and Sharjah (14.9%). In the 6- to 7-year-old group, the highest asthma prevalence was observed in Dubai (12.9%). The highest prevalence of sneezing (48.8%) and itchy rash (43.3%) was observed in Ajman. In terms of atopic dermatitis, the highest prevalence was observed in Umm Al Quwain (18.9%). In the 13- to 14-year-old group, the highest prevalence of asthma was observed in Ras Al Khaimah (12.4%). The highest prevalence of sneezing (55.3%), itchy rash (25.5%), and atopic dermatitis (18.9%) was observed in Sharjah.

## Discussion

In this study, we aimed to evaluate the prevalence and severity of asthma (wheezing) and allergies (rhinitis and atopic dermatitis) in children aged 6–7 and 13–14 years in Dubai and the Northern Emirates. Out of 3436 children who were included in our analysis, we found that the prevalence of asthma was 11.9% and 9.8% among children aged 6–7 and 13–14 years, respectively. Notably, we found that Arab ethnicity was an independent predictor of asthma occurrence. We also found that nearly 44% and 33% of children aged 6–7 and 13–14 years had experienced occurrence of wheeze or whistling at some point in their life, respectively. The prevalence of allergic rhinitis-related symptoms was 46.5% in the 6- to 7-year-old group and 51.3% for the 13- to 14-year-old group. The prevalence of allergic dermatitis-related symptoms was almost one-third in the 6- to 7-year-old group and one-quarter in the 13- to 14-year-old group.

Our analysis showed that the prevalence of asthma was higher in the 6- to 7-year-old group than in the 13- to 14-year-old group (11.9% vs. 9.8%, respectively). This low prevalence was comparable to that in the previous study of Al-Maskari et al who collected data from school-aged children (6–13 years old) in the UAE for 1997–1998 and reported a 13% prevalence of physician-diagnosed asthma.^25^ In 2006, Al-Hammadi and his colleagues demonstrated that asthma prevalence was 13.6% in the Al-Ain district among school children aged 6–9 years.^26^ These findings were supported by other reports from Kuwait,^27^ Oman,^28^^,^^29^ Egypt,^30^ Morocco, and Tunisia^31^ from 2000 to 2017. Notably, in the Saudi population, the prevalence of physician-diagnosed asthma ranged from 19.6 to 27.5% from 2000 to 2003. Such higher prevalence rates may be attributed to the difference in age group, as the Saudi study recruited participants aged 7–19 years old.^32^^,^^33^ In another report from Saudi Arabia, the prevalence of symptoms suggestive of a history of asthma was 23.6% among children 6–8 years old.^34^

Regarding the prevalence of any history of wheezing and wheezing in the past year, our findings showed a higher prevalence than the previous Emirati study of Al-Maskari et al^25^ who reported a lower prevalence of wheezing (15.6%). A similar prevalence of wheeze in the past 12 months was reported in Tunisia (13.2%) among schoolchildren aged 13–14 years during the period from 2000 to 2003.^35^ Moreover, the study of Behbehani and his colleagues reported a lower prevalence of any history of wheeze and wheeze in the past 12 months in 3- and 4-year-old children (25.9% and 16.1%, respectively), with a higher prevalence in boys than in girls (p < 0.01).^19^ In our study, there was no significant difference between both sexes in terms of wheeze in the 6- to 7-year-old group; however, in the 13- to 14-year-old group, the prevalence was higher in boys than in girls. Interestingly, we found a notable gap between the reported prevalence of asthma and wheezing in both groups, which means that many children were symptomatic but not diagnosed with asthma, indicating the importance of creating organized campaigns that aim to identify all asthmatic children. The difference between the prevalence of wheeze in our study and that in the study by Behbehani et al may be explained by the different populations and age groups of their included children. Nonetheless, our findings should be interpreted cautiously due to possible existence of information bias. Our study showed that wheezing disturbed sleep in 13.8% and 15.8% in the 6- to 7- and 13- to 14-year old groups, respectively, and speech limiting was observed in 5.1% in the 6- to 7-year-old group and 7.8% in the 13- to 14-year-old group. In contrast, disturbed sleep and speech limiting was observed in 1.5% and 1.2% among 10-year-old Omani school children.^11^

In terms of allergic rhinitis, our findings agreed with those of Behbehani and his colleagues, who reported that the prevalence of any history of allergic rhinitis, current symptoms of allergic rhinitis, and diagnosis of allergic rhinitis was 43.9%, 30.7%, and 17.1%, respectively.^19^ Another ISAAC Phase I study conducted in Saudi Arabia in 2015 by the Mahnashi et al team reported a 27.1% prevalence for allergic rhinitis among school children.^8^ In another 2008 report from Saudi Arabia, the prevalence of symptoms suggestive of a history of rhinitis was 24.2% among children aged 6–8 years.^34^ However, the prevalence of allergic rhinitis in Kuwait was 9.0%^27^ and 7.4% in Oman.^28^ Similarly, a multicenter study that involved 5 Middle Eastern countries (Egypt, Lebanon, Saudi Arabia, Iran, and the UAE), in which the data were collected in 2011, reported that the prevalence of allergic rhinitis among the Middle East population was 10%.^36^ We used the self-reporting method to assess allergic symptoms. In contrast, Ziyab et al, Al-Riyami et al, and Abdulrahman et al used a different method (physician diagnosis).^27^^,^^28^^,^^36^ In terms of allergic rhinitis severity, Mahnashi and his team showed that 12.1% had nasal block resulting in breathing difficulties, 7.8% had nasal obstruction interfering with daily activities, 6.9% had nasal obstruction, and 4.7% had disturbed sleep due to nasal blockage/problems.^8^ These percentages are similar to our findings.

In both groups, we reported a low prevalence of atopic dermatitis, which was comparable to that in the study of Al-Hammadi et al^26^ and Behbehani et al,^19^ who demonstrated that the prevalence of diagnosed atopic dermatitis was 9% and 12%, respectively. Moreover, our finding regarding itchy rash during the past year was similar to that of Al-Hammadi et al^26^ and Behbehani et al^19^ The rashes affected the elbows; behind the knees; in front of the ankles; under the buttocks; and around the neck, ears, or eyes, requiring a prompt response to overcome the resultant discomfort.

It was previously shown that environmental factors may account for the increased risk of allergic disorders in the Middle East.^37^ For example, bakhor incense was reported to affect more than half of asthmatic children (58.4%) in the study by Al-Riyami et al. Moreover, they reported that bakhor caused worsening of wheeze in 38% of asthmatics.^11^ Although there were significant positive associations between exposure to incense and any history of asthma at the bivariate level, the association lost significance at the multivariate level. This could be due to adjusting for ethnicity because the use of incense was much more prevalent among Arab than in Asian households (72.3% vs. 24.5%). It was reported that 94% of Emirati national households use bakhor on a weekly basis. In contrast, few Asians are exposed to bakhor incense, limited to exposure in some religious ceremonies.^38^^,^^39^ In terms of smoking, our multivariate analysis showed that paternal smoking was not associated with the prevalence or severity of asthma in both groups. Similarly, Joseph et al found no significant association between tobacco use in the household and the prevalence or severity of asthma.^40^

Genetic and ethnic factors play a major role in the risk of asthma and other allergic disorders. Concerning the Middle East region, a large Emirates cross-sectional study based on the ISAAC questionnaire showed that the prevalence of allergic rhinitis and asthma in immigrants was substantially lower than that in UAE citizens (OR, 0.53; 95% CI, 0.33–0.85).^20^ Similarly, we found that our study's Arab population had a two-fold higher prevalence of allergic conditions than that of other ethnicities. However, in the 13- to 14-year-old group, the odds of any history of asthma were 3 and 5 times higher among our Arab population and other ethnicity population, respectively, relative to the study's Asian population. At the multivariate analysis, it was shown that participants of Arab ethnicity were more likely to have asthma than those of South Asian ethnicity. This association between ethnicity and asthma has been reported in the literature; a similar association has also been reported for adults in the UAE.^20^^,^^41^^,^^42^ Nonetheless, it was recently shown that environmental factors can modulate the risk of allergic diseases in genetically susceptible individuals.^43^ For example, Koplin et al^44^ showed a significant effect of immigration on the risk of peanut allergy among Asian parents in Australia. Thus, future studies should investigate the influence of immigration on the prevalence of allergy in the UAE.

Mahnashi et al highlighted the considerable correlation between urban lifestyle, intermediate level of education, and lowland location as risk factors for allergic rhinitis occurrence and severity.^8^ Interestingly, Joseph et al^40^ found that in consanguineous families in the UAE, paternal asthma raised the risk of asthma for both boys and girls (p = 0.021 and p < 0.001, respectively). However, maternal asthma had no substantial effect on the risk of offspring asthma.

Another interesting finding in our covariate analysis was that the prevalence of any history of asthma was significantly higher among boys in the 6- to 7-year-old group than in girls (13.9% vs 10.0%, p = 0.021). This is consistent with the current body of evidence showing a well-established higher risk of asthma among boys than girls.^45^

Results from the video questionnaires were, to a large extent, very similar to those obtained in Kuwait among the same age group examined using the same questionnaires.^19^ Minimal differences might be due to the difference in the study period between the 2 studies.

Although our study did not reach the target sample size, the study was able to estimate the prevalence with good precision as reflected by the narrow CIs obtained in the results. Moreover, the study was able to test the association between all demographic and environmental factors in the study with asthma. Nonetheless, our study has some limitations. First, children in the 6- to 7-year-old group were school-age students, and the questionnaire was answered by their parents, who may have provided inaccurate answers. Second, this study depended on participant experience to report symptoms, which can be misinterpreted in the form of over/underestimating symptoms, which may affect the results of this study. Third, the correlation of environmental factors with allergic rhinitis laboratory tests should confirm related symptoms. Finally, the Arabic version of the ISAAC questionnaire was adopted from the study by Behbehan et al,^19^ which was performed in Kuwait. In this study, the Arabic questionnaire was validated based on expert consensus alone. Although this validation method has certain limitations, a previous report by the ISAAC phase III group noted that repeated ISAAC studies are feasible and achievable, using multiple translations made by people with diverse cultural backgrounds.^46^ Many of these translated versions were validated using a method similar to that used by Behbehan et al.^19^

In conclusion, the prevalence of asthma, wheezing, and atopic dermatitis was comparable to that reported in the previous literature in the UAE and neighboring countries (Kuwait, Oman, and Saudi Arabia). Relative to Asian participants, Arab participants had a significantly higher prevalence of any history of asthma. Overall, the prevalence of any history of asthma was highest in Ras Al Khaimah, followed by Dubai, and the lowest prevalence was in Ajman. There was no significant correlation between exposure to parental tobacco use and/or incense and asthma prevalence. Patients with asthma with allergic rhinitis had more severe respiratory symptoms than those without allergic rhinitis. These findings suggest that more efforts are needed to organize nationwide campaigns to identify undiagnosed children to reduce the burden of these conditions.

## Abbreviations

CI: confidence interval; ISAAC: International Study of Asthma and Allergies in Childhood; KDHA: Knowledge & Human Development Authority; MOE: Ministry of Education; MOHP: Ministry of Health and Prevention; OR: odds ratio; STROBE: Strengthening the Reporting of Observational Studies in Epidemiology; UAE: United Arab Emirates; WHO: World Health Organization.

## Ethics, consent and permissions

The study was approved by the Ministry of Health and Prevention (MOHP), UAE (Ref No. MOHAP/DXB-REC-6/FM/2019), and the participants signed written informed consent.

## Availability of data and material

Not applicable.

## Author contributions

**Nassem Mohamed Ibrahim: (1)** made substantial contributions to conception and design and interpretation of data; (2) reviewed the article critically for important intellectual content; (3) given final approval of the version to be published; and (4) agrees to be accountable for all aspects of the work related to its accuracy or integrity.

**Fatima Ibrahim Almarzouqi:** made substantial contributions to conception, design, and interpretation of data; (2) drafted the article; (3) given final approval of the version to be published; and (4) agrees to be accountable for all aspects of the work related to its accuracy or integrity.

**Fatima Abdulla Al Melaih:** made substantial contributions to acquisition of data; (2) drafted the article; (3) given final approval of the version to be published; and (4) agrees to be accountable for all aspects of the work related to its accuracy or integrity.

**Hisham Farouk:** made substantial contributions to data analysis and interpretation of data; (2) drafted the article; (3) given final approval of the version to be published; and (4) agrees to be accountable for all aspects of the work related to its accuracy or integrity.

**Mohamed Alsayed:** made substantial contributions to data analysis and interpretation of data; (2) drafted the article; (3) given final approval of the version to be published; and (4) agrees to be accountable for all aspects of the work related to its accuracy or integrity.

**Fatma Mohamed AlJassim:** made substantial contributions to conception and design; (2) reviewed the article critically for important intellectual content; (3) given final approval of the version to be published; and (4) agrees to be accountable for all aspects of the work related to its accuracy or integrity.

## Submission declaration

We confirm that the present manuscript has not been published previously and it is not under consideration for publication elsewhere. Its publication is approved by all authors and tacitly or explicitly by the responsible authorities where the work was carried out, and that, if accepted, it will not be published elsewhere in the same form, in English or in any other language, including electronically without the written consent of the copyright holder.

## Declaration of competing interest

This work was supported by 10.13039/100004325AstraZeneca, who funded the study. Representatives of the sponsor were study design, data gathering, analysis, interpretation, and revision. Hisham Farouk and Mohamed Alsayed work for AstraZeneca; however, there is no conflict of interest. The rest of the authors declare that they have no conflict of interest.
