# Supplementary material for: Prevalence of asthma and allergies among children in the United Arab Emirates: A cross-sectional study
Source: World Allergy Organ J. 2021 Oct 6;14(10):100588. doi: 10.1016/j.waojou.2021.100588 (PMC8503660; doi:10.1016/j.waojou.2021.100588)
Supplement: Multimedia component 1 [file mmc1.docx]

eTable 1: Results of the video showing

| Variable | n | % | 95% Confidence Interval |
| --- | --- | --- | --- |
| Wheeze at rest ever |  |  |  |
| Yes | 276 | 17.1% | (15.3%, 19.0%) |
| No | 1334 | 82.9% |  |
| Wheeze at rest in the last year |  |  |  |
| Yes | 203 | 12.6% | (11.0%, 14.2%) |
| No | 1406 | 87.4% |  |
| Wheeze at rest ≥ 1 per month |  |  |  |
| Yes | 113 | 7.1% | (5.8%. 8.4%) |
| No | 1477 | 92.9% |  |
| Wheeze during exercise ever |  |  |  |
| Yes | 296 | 18.5% | (16.6%, 20.3%) |
| No | 1306 | 81.5% |  |
| Wheeze during exercise in the last year |  |  |  |
| Yes | 234 | 14.7% | (13.0%, 16.4%) |
| No | 1357 | 85.3% |  |
| Wheeze during exercise ≥1 per month |  |  |  |
| Yes | 142 | 9.0% | (7.6%, 10.4%) |
| No | 1432 | 91.0% |  |
| Nocturnal Wheeze ever |  |  |  |
| Yes | 125 | 7.8% | (6.5%, 9.1%) |
| No | 1473 | 92.2% |  |
| Nocturnal Wheeze in the last year |  |  |  |
| Yes | 99 | 6.2% | (5.0%, 7.4%) |
| No | 1494 | 93.8% |  |
| Nocturnal Wheeze ≥1 per month |  |  |  |
| Yes | 58 | 3.7% | (2.7%, 4.6%) |
| No | 1529 | 96.3% |  |
| Nocturnal cough ever |  |  |  |
| Yes | 410 | 25.5% | (23.4%, 27.6%) |
| No | 1198 | 74.5% |  |
| Nocturnal cough in the last year |  |  |  |
| Yes | 302 | 18.9% | (17.0%, 20.8%) |
| No | 1294 | 81.1% |  |
| Nocturnal cough ≥1 per month |  |  |  |
| Yes | 158 | 10.1% | (8.6%, 11.5%) |
| No | 1413 | 89.9% |  |
| Severe Asthma attack ever |  |  |  |
| Yes | 178 | 11.2% | (9.6%, 12.7%) |
| No | 1418 | 88.8% |  |
| Severe Asthma attack in the last year |  |  |  |
| Yes | 142 | 8.9% | (7.5%, 10.3%) |
| No | 1449 | 91.1% |  |
| Severe Asthma attack ≥1 per month |  |  |  |
| Yes | 81 | 5.1% | (4.0%, 6.2%) |
| No | 1503 | 94.9% |  |
| combined wheezing ever |  |  |  |
| Yes | 460 | 28.5% | (26.3%, 30.7%) |
| No | 1154 | 71.5% |  |
| combined wheezing in the past year |  |  |  |
| Yes | 371 | 23.0% | (20.9%, 25.0%) |
| No | 1243 | 77.0% |  |

eTable 2: Comparing video results by gender

| **Variable** |  | **Female** | | **Male** | | **p-value** |
| --- | --- | --- | --- | --- | --- | --- |
|  |  | **N** | **%** | **n** | **%** |  |
| **Wheeze at rest ever** | **No** | 817 | 83.9% | 512 | 81.3% |  |
|  | **Yes** | 157 | 16.1% | 118 | 18.7% | 0.175 |
| **Wheeze at rest in the last year** | **No** | 853 | 87.6% | 548 | 87.1% |  |
|  | **Yes** | 121 | 12.4% | 81 | 12.9% | 0.789 |
| **Wheeze at rest ≥ 1 per month** | **No** | 888 | 92.4% | 584 | 93.7% |  |
|  | **Yes** | 73 | 7.6% | 39 | 6.3% | 0.311 |
| **Wheeze during exercise ever** | **No** | 814 | 84.0% | 486 | 77.6% |  |
|  | **Yes** | 155 | 16.0% | 140 | 22.4% | 0.001* |
| **Wheeze during exercise in the last year** | **No** | 836 | 86.5% | 515 | 83.3% |  |
|  | **Yes** | 130 | 13.5% | 103 | 16.7% | 0.079 |
| **Wheeze during exercise ≥1 per month** | **No** | 878 | 91.8% | 548 | 89.7% |  |
|  | **Yes** | 78 | 8.2% | 63 | 10.3% | 0.147 |
| **Nocturnal Wheeze ever** | **No** | 890 | 92.3% | 577 | 92.0% |  |
|  | **Yes** | 74 | 7.7% | 50 | 8.0% | 0.828 |
| **Nocturnal Wheeze in the last year** | **No** | 896 | 93.1% | 592 | 94.9% |  |
|  | **Yes** | 66 | 6.9% | 32 | 5.1% | 0.162 |
| **Nocturnal Wheeze ≥1 per month** | **No** | 920 | 95.9% | 603 | 97.1% |  |
|  | **Yes** | 39 | 4.1% | 18 | 2.9% | 0.224 |
| **Nocturnal cough ever** | **No** | 702 | 72.4% | 492 | 77.8% |  |
|  | **Yes** | 268 | 27.6% | 140 | 22.2% | 0.014* |
| **Nocturnal cough in the last year** | **No** | 759 | 78.8% | 531 | 84.7% |  |
|  | **Yes** | 204 | 21.2% | 96 | 15.3% | 0.003* |
| **Nocturnal cough ≥1 per month** | **No** | 834 | 88.0% | 575 | 93.2% |  |
|  | **Yes** | 114 | 12.0% | 42 | 6.8% | 0.001* |
| **Severe Asthma attack ever** | **No** | 847 | 87.9% | 567 | 90.6% |  |
|  | **Yes** | 117 | 12.1% | 59 | 9.4% | 0.092 |
| **Severe Asthma attack in the last year** | **No** | 865 | 89.9% | 580 | 93.1% |  |
|  | **Yes** | 97 | 10.1% | 43 | 6.9% | 0.029* |
| **Severe Asthma attack ≥1 per month** | **No** | 903 | 94.3% | 595 | 96.0% |  |
|  | **Yes** | 55 | 5.7% | 25 | 4.0% | 0.131 |
| **combined wheezing ever** | **No** | 717 | 73.5% | 432 | 68.4% |  |
|  | **Yes** | 258 | 26.5% | 200 | 31.6% | 0.025* |
| **combined wheezing in the past year** | **No** | 753 | 77.2% | 485 | 76.7% |  |
|  | **Yes** | 222 | 22.8% | 147 | 23.3% | 0.819 |

*significant difference

eTable 3: Bivariate association between ever asthma and variables in the study

| **Variable** | | **6-7 years (n =1778)** | | | | | **13-14 years (n =1641)** | | | | |
| --- | --- | --- | --- | --- | --- | --- | --- | --- | --- | --- | --- |
|  |  | **No** | | **Yes** | | **P-value** | **No** | | **Yes** | | **P-value** |
|  |  | **N** | **%** | **N** | **%** |  | **N** | **%** | **N** | **%** |  |
| **Ever Sneezing** | No | 726 | 91.0% | 72 | 9.0% | <0.001* | 683 | 92.9% | 52 | 7.1% | <0.001* |
|  | Yes | 580 | 84.9% | 103 | 15.1% |  | 682 | 87.8% | 95 | 12.2% |  |
| **Ever Wheezing** | No | 741 | 94.4% | 44 | 5.6% | <0.001* | 948 | 95.5% | 45 | 4.5% | <0.001* |
|  | Yes | 576 | 81.4% | 132 | 18.6% |  | 418 | 80.2% | 103 | 19.8% |  |
| **Ever hay fever** | No | 1012 | 93.8% | 67 | 6.2% | <0.001* | 1067 | 92.5% | 86 | 7.5% | <0.001* |
|  | Yes | 212 | 70.2% | 90 | 29.8% |  | 233 | 81.5% | 53 | 18.5% |  |
| **Ever Eczema** | No | 1084 | 92.5% | 88 | 7.5% | <0.001* | 1114 | 91.9% | 98 | 8.1% | <0.001* |
|  | Yes | 116 | 68.2% | 54 | 31.8% |  | 173 | 83.2% | 35 | 16.8% |  |
| **Ever Itchy Rash** | No | 854 | 89.1% | 104 | 10.9% | 0.134 | 1020 | 91.2% | 98 | 8.8% | 0.056 |
|  | Yes | 449 | 86.5% | 70 | 13.5% |  | 342 | 87.9% | 47 | 12.1% |  |
| **School Type** | Private | 1043 | 89.4% | 124 | 10.6% | 0.005* | 892 | 91.5% | 83 | 8.5% | 0.029* |
|  | Public | 279 | 83.8% | 54 | 16.2% |  | 477 | 88.0% | 65 | 12.0% |  |
| **School City** | Ajman | 133 | 94.3% | 8 | 5.7% | 0.111 | 133 | 91.1% | 13 | 8.9% | 0.699 |
|  | Dubai | 445 | 87.4% | 64 | 12.6% |  | 481 | 90.1% | 53 | 9.9% |  |
|  | Fujairah | 131 | 85.1% | 23 | 14.9% |  | 198 | 93.0% | 15 | 7.0% |  |
|  | Ras Al Khaimah | 126 | 86.3% | 20 | 13.7% |  | 138 | 88.5% | 18 | 11.5% |  |
|  | Sharjah | 454 | 88.8% | 57 | 11.2% |  | 388 | 89.6% | 45 | 10.4% |  |
|  | Umm Al Quawain | 33 | 84.6% | 6 | 15.4% |  | 31 | 88.6% | 4 | 11.4% |  |
| **City of residence** | Abu Dhabi | 1 | 100.0% | 0 | 0.0% | 0.688 | 1 | 100.0% | 0 | 0.0% | 0.707 |
|  | Ajman | 154 | 92.2% | 13 | 7.8% |  | 141 | 92.8% | 11 | 7.2% |  |
|  | Dubai | 406 | 87.1% | 60 | 12.9% |  | 434 | 89.9% | 49 | 10.1% |  |
|  | Fujairah | 100 | 89.3% | 12 | 10.7% |  | 187 | 92.1% | 16 | 7.9% |  |
|  | Ras Al Khaimah | 123 | 87.9% | 17 | 12.1% |  | 113 | 87.6% | 16 | 12.4% |  |
|  | Sharjah | 433 | 88.7% | 55 | 11.3% |  | 427 | 89.7% | 49 | 10.3% |  |
|  | Umm Al Quawain | 31 | 88.6% | 4 | 11.4% |  | 34 | 91.9% | 3 | 8.1% |  |
| **Ethnicity** | Arab | 703 | 83.4% | 140 | 16.6% | <0.001* | 807 | 88.0% | 110 | 12.0% | <0.001* |
|  | Others | 126 | 90.0% | 14 | 10.0% |  | 59 | 80.8% | 14 | 19.2% |  |
|  | South Asians | 443 | 95.9% | 19 | 4.1% |  | 492 | 95.5% | 23 | 4.5% |  |
| **Father Smoking** | No | 1058 | 89.7% | 122 | 10.3% | <0.001* | 1080 | 91.1% | 105 | 8.9% | 0.023* |
|  | Yes | 259 | 82.5% | 55 | 17.5% |  | 286 | 86.9% | 43 | 13.1% |  |
| **Mother smoking** | No | 1296 | 88.2% | 174 | 11.8% | 0.304 | 1320 | 90.1% | 145 | 9.9% | 0.619 |
|  | Yes | 17 | 81.0% | 4 | 19.0% |  | 45 | 93.8% | 3 | 6.3% |  |
| **Exposure to incense** | No | 784 | 91.3% | 75 | 8.7% | <0.001* | 415 | 93.0% | 31 | 7.0% | 0.018* |
|  | Yes | 499 | 83.3% | 100 | 16.7% |  | 947 | 89.1% | 116 | 10.9% |  |
| Gender | Female | 690 | 90.0% | 77 | 10.0% | 0.021* | 832 | 91.3% | 79 | 8.7% | 0.104 |
|  | Male | 625 | 86.1% | 101 | 13.9% |  | 531 | 88.8% | 67 | 11.2% |  |

eTable 4: Multivariate logistic regression model for independent predictors of ever asthma.

| **Variable** | | **6-7 years (n =1778)** | | | | **13-14 years (n =1641)** | | | |
| --- | --- | --- | --- | --- | --- | --- | --- | --- | --- |
|  |  | **ARR** | **95% C.I. For ARR** | | **P-value** | **ARRR** | **95% C.I. for ARR** | | **P-value** |
|  |  |  | **Lower** | **Upper** |  |  | **Lower** | **Upper** |  |
| **Ever Sneezing vs. never sneezing** | | 0.993 | 0.627 | 1.543 | 0.977 | 1.193 | 0.783 | 1.789 | 0.406 |
| **Ever Wheezing vs. never wheezing** | | 3.575 | 2.375 | 5.179 | <0.001* | 4.881 | 3.439 | 6.711 | <0.001* |
| **Ever hay fever vs. never hay fever** | | 3.478 | 2.428 | 4.823 | <0.001* | 2.035 | 1.389 | 2.910 | <0.001* |
| **Ever Eczema vs. never eczema** | | 3.263 | 2.177 | 4.663 | <0.001* | 1.829 | 1.183 | 2.740 | 0.007* |
| **Ever Itchy Rash vs. never itchy rash** | | 0.577 | 0.356 | 0.922 | 0.021* | 0.678 | 0.430 | 1.030 | 0.069 |
| **Private vs. public schools** | | 0.815 | 0.511 | 1.259 | 0.367 | 1.134 | 0.733 | 1.720 | 0.567 |
| **School City** | Ajman | 0.520 | 0.105 | 2.043 | 0.384 | NA | | | |
|  | Dubai | 1.622 | 0.526 | 3.617 | 0.373 |  |  |  |  |
|  | Fujairah | 1.242 | 0.365 | 3.147 | 0.710 |  |  |  |  |
|  | Ras Al Khaimah | 0.960 | 0.265 | 2.695 | 0.947 |  |  |  |  |
|  | Sharjah | 1.030 | 0.311 | 2.688 | 0.959 |  |  |  |  |
|  | Umm Al Quawain | 1 |  | | |  |  |  |  |
| **Ethnicity** | Arab | 2.161 | 1.184 | 3.811 | 0.012* | 2.790 | 1.636 | 4.577 | <0.001* |
|  | Others | 1.518 | 0.686 | 3.222 | 0.299 | 4.368 | 2.220 | 7.791 | <0.001* |
|  | South Asians | 1 |  | | | 1 |  | | |
| **Father Smoking vs. father not smoking** | | 1.207 | 0.795 | 1.790 | 0.370 | 1.370 | 0.913 | 2.012 | 0.127 |
| **Exposure to incense vs. no exposure to incense** | | 1.093 | 0.712 | 1.646 | 0.681 | 1.160 | 0.737 | 1.793 | 0.517 |
| **Female Gender** | | 0.810 | 0.556 | 1.158 | 0.253 | 0.770 | 0.531 | 1.102 | 0.156 |

*significant difference in the ever asthma after adjusting for the rest of the variables in the model presented

eTable 5: The prevalence of Wheeze Ever, Last Year Wheeze, and Last Year Nocturnal Wheeze: Concordance between the ISAAC Written and Video Questionnaires

| **Questionnaire** | | | **Wheeze Ever** | | **Wheeze in the last year** | | **Nocturnal Wheeze in the last year** | |
| --- | --- | --- | --- | --- | --- | --- | --- | --- |
| **a** | | | | | | | | |
| **Written** | | **Video** | n | % | n | % | n | % |
| **No** | | **no** | 977 | 61% | 1056 | 66% | 1311 | 90% |
| **Yes** | | **yes** | 184 | 11% | 121 | 8% | 43 | 3% |
| **Yes** | | **no** | 352 | 22% | 338 | 21% | 53 | 4% |
| **No** | | **yes** | 92 | 6% | 82 | 5% | 51 | 3% |
| **Concordance** | | | 1161 | 72% | 1177 | 74% | 1354 | 93% |
| **Percentage of agreement** | | | P |  | P |  | P |  |
| **Negative responses** | | | 69% |  | 72% |  | 93% |  |
| **positive responses** | | | 29% |  | 22% |  | 29% |  |
| **Kappa** | | | 0.29 |  | 0.23 |  | 0.41 |  |
| **b** | | | | | | | | |
| **Written** | **Video** | | n | % | n | % |  |  |
| **No** | **no** | | 870 | 54% | 952 | 60% |  |  |
| **Yes** | **yes** | | 259 | 16% | 181 | 11% |  |  |
| **Yes** | **no** | | 279 | 17% | 279 | 17% |  |  |
| **No** | **yes** | | 201 | 13% | 189 | 12% |  |  |
| **Concordance** | | | 1129 | 70% | 1133 | 71% |  |  |
| **Percentage of agreement** | | | P |  | P |  |  |  |
| **Negative responses** | | | 64% |  | 67% |  |  |  |
| **positive responses**  **Kappa** | | | 35%  0.31 |  | 28%  0.24 |  |  |  |

aThe response to the first video (wheeze at rest) is considered, P=percentage of agreement.

bThe response of yes in the video is defined as any yes for the three videos: wheeze at rest, during exercise or nocturnal wheeze), P=percentage of agreeme
